# Supplementary material for: Methodological characteristics of Peruvian clinical practice guidelines, 2018 – 2023: A scoping review
Source: PLoS One. 2025 Dec 31;20(12):e0339861. doi: 10.1371/journal.pone.0339861 (PMC12755755; doi:10.1371/journal.pone.0339861)
Supplement: S1 Table — (DOCX) [file pone.0339861.s001.docx]

## **S1 Table. Search strategies**

| **Search Engine, Repository, or Local Organization** | | **Link** | **Observations** | **CPGs that met inclusion criteria** |
| --- | --- | --- | --- | --- |
| **Local Organizations** | | | | |
| Social Security Health Insurance (EsSalud) | Institute of Health Technology Assessment and Research of the Social Security Health Insurance (IETSI) | https://ietsi.essalud.gob.pe/gpc-guias-de-practica-clinica/ | None | 53 |
| AUNA Peru Network | Oncosalud | <https://www.oncosalud.pe/direccion-cientifica-academica/guias-de-practica-clinica-auna> | None | 8 |
| Ministry of Health (MINSA) | Unit for Analysis and Evidence Generation in Public Health (UNAGESP) of the National Institute of Health (INS) | https://www.gob.pe/institucion/ins/informes-publicaciones/tipos/217-guias-de-practica-clinica | None | 8 |
|  |  | https://repositorio.ins.gob.pe/handle/20.500.14196/255 | Filtered by “guía de práctica clínica” |  |
|  | National Children's Health Institute of San Borja (INSNSB) | <https://www.insnsb.gob.pe/guias-de-practica-clinicas/> | None | 1 |
|  | National Maternal Perinatal Institute (INMP) | https://www.inmp.gob.pe/institucional/guias-de-practica-clinica-y-de-procedimiento-del-departamento-en-neonatologia/1680205704 | None | 0 |
|  |  | https://www.inmp.gob.pe/institucional/guias/1590593033 | None |  |
|  | Víctor Larco Herrera Hospital (HVLH) | <https://larcoherrera.gob.pe/resoluciones-hvlh-elei/> | Director’s Resolutions approving Clinical Practice Guidelines from 2018 to 2023 were reviewed | 0 |
|  | National Institute of Neoplastic Diseases (INEN) | <https://portal.inen.sld.pe/guias-tecnicas/> | None | 5 |
|  | National Institute of Neurological Sciences (INCN) | https://www.incn.gob.pe/guias-de-practica-clinica-y-consentimientos-informados/ | None | 2 |
|  | Single Digital Platform of the Peruvian State - Ministry of Health | https://www.gob.pe/minsa | “Guía Práctica Clínica” was searched. Filtered by “Tipos de Contenido: Normas y Documentos Legales, Subtipo: Resoluciones”, Publication Date: from 01/01/2018 | 2 |
|  |  |  | “Guía Práctica Clínica” was searched. Filtered by “Tipos de Contenido: Informes y publicaciones, Subtipo: Publicaciones”, Publication Date: from 01/01/2018 |  |
|  |  |  | “Guía Práctica Clínica” was searched. Filtered by “Tipos de Contenido: Informes y publicaciones, Subtipo: Guías”, Publication Date: from 01/01/2018 |  |
|  | National Institute of Ophthalmology (INO) | https://www.ino.gob.pe/documentos-tecnicos/ | None | 6 |
|  | National Institute of Mental Health "Honorio Delgado - Hideyo Noguchi" | https://www.insm.gob.pe/transparencia/normaslegales.html | Director’s Resolutions were reviewed | 0 |
|  |  | https://www.insm.gob.pe/departamentos/emergencia/docencia.html | None |  |
| **Search Engines** | | | | |
| Google | | https://www.google.com/ | “(guia practica clinica OR recomendaciones) AND (peru)”  The appropriate date filter was used | 0 |
| Google Scholar | | https://scholar.google.com.pe/ | “(guia practica clinica OR recomendaciones) AND (peru)”  The appropriate date filter was used. Sorted by relevance. | 3 |
