# Supplementary material for: Methodological characteristics of Peruvian clinical practice guidelines, 2018 – 2023: A scoping review
Source: PLoS One. 2025 Dec 31;20(12):e0339861. doi: 10.1371/journal.pone.0339861 (PMC12755755; doi:10.1371/journal.pone.0339861)
Supplement: S2 Table — (DOCX) [file pone.0339861.s002.docx]

## **S2 Table. Table of excluded CPGs**

| **N°** | **Organization** | **Name of the CPG** | **Year** | **Reason for exclusion** |
| --- | --- | --- | --- | --- |
| 1 | IETSI | Guía de Práctica Clínica para el manejo del paciente con Angina Estable | 2023 | Partial update of a previous CPG |
| 2 | IETSI | Guía de Práctica clínica para el manejo de pacientes con Fibrilación Auricular | 2023 | Partial update of a previous CPG |
| 3 | INMP | Guías de Práctica Clínica y de procedimientos en Obstetricia y Perinatología del 2018 | 2018 | No full-text version with public access on the web |
| 4 | INSNSB | Guía de Práctica Clínica de Diagnóstico y Tratamiento del Síndrome de Noonan | 2020 | No full-text version with public access on the web |
| 5 | MINSA | Guía de Práctica Clínica para Diagnóstico y tratamiento de Bronquiolitis en niños menores de dos años | 2019 | No full-text version with public access on the web |
| 6 | MINSA | Guía de práctica clínica para el diagnóstico y tratamiento de la neumonía en la niña y el niño | 2019 | No full-text version with public access on the web |
| 7 | MINSA | Guía de Práctica Clínica para Tamizaje, Diagnóstico y Tratamiento de la Depresión en Personas Mayores de 18 años desde el Primer Nivel de Atención | 2023 | No full-text version with public access on the web |
| 8 | MINSA | Guía de Práctica Clínica de Diagnóstico y Tratamiento de Diabetes Mellitus Tipo No Insulinodependiente (Tipo 2) con Cetoacidosis, Estado Hiperglicémico Hiperosmolar y Estado Mixto | 2023 | No full-text version with public access on the web |
| 9 | MINSA | Guía de Práctica Clínica para el Diagnóstico y Tratamiento de la Diabetes Mellitus tipo 1 en niños, adolescentes y adultos | 2024 | No full-text version with public access on the web |
| 10 | AUNA Peru Network | Guía de Práctica Clínica de manejo multidisciplinario de melanoma cutáneo | 2021 | No full-text version with public access on the web |
| 11 | AUNA Peru Network | Guía de Práctica Clínica de cáncer de tiroides | 2021 | No full-text version with public access on the web |
| 12 | AUNA Peru Network | MANEJO DE LAS CRISIS GLUCÉMICAS EN PACIENTES ADULTOS CON DIABETES MELLITUS: GUÍA DE PRÁCTICA CLÍNICA BASADA EN EVIDENCIAS | 2021 | No full-text version with public access on the web |
| 13 | AUNA Peru Network | Manejo multidisciplinario de la Neutropenia Febril: Consenso Institucional. Lima, Perú | 2021 | No full-text version with public access on the web |
| 14 | AUNA Peru Network | Guía de Práctica Clínica para el Manejo del Dolor en pacientes Oncológicos de una Red de Clínicas Privadas, Lima - Perú | 2022 | No full-text version with public access on the web |
| 15 | AUNA Peru Network | Manejo Multidisciplinario del Adenocarcinoma de Páncreas: Guía de Práctica Clínica AUNA | 2022 | No full-text version with public access on the web |
| 16 | AUNA Peru Network | Guía de Práctica Clínica de manejo multidisciplinario del cáncer de mama | 2020 | Partial update of a previous CPG |
| 17 | AUNA Peru Network | Guía de Práctica Clínica de manejo multidisciplinario de cáncer de próstata | 2019 | Partial update of a previous CPG |
| 18 | Peruvian Society of Oncology | Diagnóstico y tratamiento del cáncer de mama HER2+: Guía de Práctica Clínica de la Sociedad Peruana de Cancerología | 2020 | No full-text version with public access on the web |

IETSI - EsSalud: Institute of Health Technology Assessment and Research of the Social Security Health Insurance; INMP: National Maternal Perinatal Institute; INSNSB: National Children’s Health Institute of San Borja; MINSA: Ministry of Health.
