# Supplementary material for: Methodological characteristics of Peruvian clinical practice guidelines, 2018 – 2023: A scoping review
Source: PLoS One. 2025 Dec 31;20(12):e0339861. doi: 10.1371/journal.pone.0339861 (PMC12755755; doi:10.1371/journal.pone.0339861)
Supplement: Attachment — Submitted filename: Response to reviewers.docx [file pone.0339861.s006.docx]

**PONE-D-24-51671**
**Title:** Methodological characteristics of Peruvian clinical practice guidelines, 2018 - 2023: a scoping review

**Response letter**

Dear Editor and reviewers,

Thank you for your kind consideration. In this letter, we proceed to respond to the editor’s and reviewers’ comments.

Sincerely,

Alvaro Taype-Rondan, corresponding author

**Editor’s comments:**

1. You should compile data about previous efforts to standardize Clinical Practice Guidelines (CPGs) in Peru and other South American countries. Regarding discussion and conclusions, what actions should be expected in the following years? How could this study contribute to improving CPG management in Peru? Please add these topics in the discussion.

Answer: Thanks for your comment. We have added the following information: “Efforts to standardize CPG development in Latin America have employed various strategies, gradually shifting toward evidence-based practices since 2014 [30]. This shift, driven by academic institutions and research groups, has prioritized guideline adaptation, adoption, or de novo development, supported by the creation of methodological manuals [30]. This approach has gained traction across the region, with countries like Brazil [31], Colombia [32], Chile [13], Mexico [5], Argentina [12], and Peru [9] establishing standards for both CPG development and implementation of CPGs [30]. In Peru, the first technical document, issued in 2005, classified evidence using the U.S. Preventive Services Task Force model but lacked robust processes for clinical question formulation or guideline adaptation [33]. The 2015 technical document marked progress by incorporating PICO (Population, Intervention, Comparator, Outcome) questions, systematic reviews, and the GRADE approach [9]. Despite improvements, challenges persist in ensuring the consistent production and implementation of evidence-based CPGs.

Improving CPG management in Peru should focus on enhancing the training of methodologists in evidence synthesis, increasing the integration of economic analysis in decision-making, and ensuring greater consistency in guideline development processes. In the coming years, expected actions include greater collaboration between institutions, strengthening oversight mechanisms, and the continued development of robust training programs for CPG developers. Our study contributes to ongoing discussions on improving CPG quality and implementation in Peru by providing a snapshot of current trends and identifying critical gaps that need to be addressed to enhance the evidence base for healthcare decision-making.”

**Journal Requirements:**

1. Please ensure that your manuscript meets PLOS ONE's style requirements, including those for file naming. The PLOS ONE style templates can be found at
   <https://journals.plos.org/plosone/s/file?id=wjVg/PLOSOne_formatting_sample_main_body.pdf> and 
   <https://journals.plos.org/plosone/s/file?id=ba62/PLOSOne_formatting_sample_title_authors_affiliations.pdf>

Answer: We verified that our manuscript follows PLOS ONE’s style requirements.

2. Thank you for stating the following in the Competing Interests section:
   I have read the journal's policy and the authors of this manuscript have the following competing interests: ABC, JRM, LCR and ATR have participated in the development of clinical practice guidelines for the Institute of Health Technology Assessment and Research (IETSI) of EsSalud and have received compensation for their work. The authors declare no additional potential conflicts of interest related to this study.

Please confirm that this does not alter your adherence to all PLOS ONE policies on sharing data and materials, by including the following statement: ""This does not alter our adherence to  PLOS ONE policies on sharing data and materials.” (as detailed online in our guide for authors <http://journals.plos.org/plosone/s/competing-interests>).  If there are restrictions on sharing of data and/or materials, please state these. Please note that we cannot proceed with consideration of your article until this information has been declared.

Please include your updated Competing Interests statement in your cover letter; we will change the online submission form on your behalf.

Answer: We have updated the competing interests section and included it in the cover letter: “ABC, JRM, LCR and ATR have participated in the development of clinical practice guidelines for the Institute of Health Technology Assessment and Research (IETSI) of EsSalud and have received compensation for their work. The authors declare no additional potential conflicts of interest related to this study. This does not alter our adherence to PLOS ONE policies on sharing data and materials”.

**Reviewers’ comments:**

**R2.1:** Please convert all dataset variable names from Spanish to English, as shown in the provided Excel file, to improve accessibility for international researchers. This translation will help external reviewers and collaborators better understand the variables and verify the data.

Answer: We changed all dataset variable names to English.

**R2.2:** The introduction mentions deficiencies in methodology found in previous studies. To provide context, consider creating a table or a brief review summarizing these past methodological shortcomings. Then, compare these with the findings from your current study. This addition would be beneficial for future researchers by highlighting patterns and supporting efforts to standardize Clinical Practice Guidelines (CPGs) in South America.

Answer: Thanks for your comment. We have added the following information to the Introduction section: “Common shortcomings include unclear processes for formulating recommendations [4-6], inadequate systematic searches and selection criteria [4,5], limited consideration of evidence strengths and limitations [4-6], and failure to incorporate patients' preferences [4,6]. In Peru, an evaluation of 17 CPGs published by the Ministry of Health between 2009 and 2014 revealed similar deficiencies [7], including a lack of information about the guideline development team, minimal use of evidence, and unclear recommendation processes [7]. A further study of 31 CPGs developed by the Peruvian Social Health Insurance System (EsSalud) up to December 2015 reported gaps in evidence-based frameworks and inconsistent reporting of recommendations [8].”

Also, we have added the following information to the Discussion section: “This shortfall may be attributed to a lack of trained methodologists in GRADE methodology, as reported in other countries in the region, such as Brazil [22], In Mexico, however, the adherence to the GRADE methodology was even lower, with only 29.1% of CPGs claiming to use it, which highlights an even greater issue in guideline implementation [5]. This issue has been similarly identified in reviews of health technology assessments in Peru (4.6%) [23].”

**R2.3:** What are the standards for CPGs in Peru versus other countries? Provide an overview of international standards for CPGs, if available, and discuss the differences with Peruvian standards. Consider sharing your perspective on what constitutes an ideal standard for CPGs, with a critical analysis. For instance, how might the CPGs from countries like Japan or the U.S. be considered superior, and what aspects could be improved?

Answer: Thanks for your comment. We have added the following information in the Introduction section: “Internationally, institutions such as the World Health Organization (WHO) [10], National Institute for Health and Care Excellence (NICE) in the UK [11], and regional organizations such as the Ministry of Health of Argentina [12] and Chile [13] follow rigorous methodologies emphasizing transparency, evidence quality assessment, and regular updates. While Peru's standards have advanced, they could benefit from greater alignment with these international practices, ensuring guidelines remain evidence-based and relevant.”

We also have added information in the Discussion section: “Efforts to standardize CPG development in Latin America have employed various strategies, gradually shifting toward evidence-based practices since 2014 [30]. This shift, driven by academic institutions and research groups, has prioritized guideline adaptation, adoption, or de novo development, supported by the creation of methodological manuals [30]. This approach has gained traction across the region, with countries like Brazil [31], Colombia [32], Chile [13], Mexico [5], Argentina [12], and Peru [9] establishing standards for both CPG development and implementation of CPGs [30]. In Peru, the first technical document, issued in 2005, classified evidence using the U.S. Preventive Services Task Force model but lacked robust processes for clinical question formulation or guideline adaptation [33]. The 2015 technical document marked progress by incorporating PICO (Population, Intervention, Comparator, Outcome) questions, systematic reviews, and the GRADE approach [9]. Despite improvements, challenges persist in ensuring the consistent production and implementation of evidence-based CPGs.

Improving CPG management in Peru requires a multi-faceted approach, including increased investment in training programs for methodologists, the creation of a central oversight body for quality assurance, and collaboration with international partners to adopt best practices. Future efforts should also focus on expanding access to data for economic evaluations and developing tailored tools to facilitate systematic resource use assessments.”

**R2.4:** Expand on the data collection process, specifically the consensus method. Include details on how consensus was reached, the criteria used, and the process undertaken to ensure quality in data collection. This additional information would help demonstrate the rigor applied to maintain data reliability.

Answer: Thanks for your comment. We added the following information: “In case of disagreements, consensus was reached through discussion among the investigators, followed by a second review of the data from the included CPGs to identify the precise information. To ensure the quality of the data collection process, a predefined set of criteria was applied to each variable. This included cross-checking the extracted information with the original documents, verifying consistency across multiple versions and journal articles of the CPGs, and ensuring the accuracy of all recorded data. If consensus could not be reached, another researcher (ATR) was consulted to resolve any uncertainties or clarify discrepancies in the data.”

**R2.5:** In Table 3, in addition to the p-values and percentages, please include the standard deviation and 95% confidence intervals for each result. This added statistical detail will provide a clearer picture of the variability and confidence in the results.

Answer: Thank you for your suggestion. However, the variables presented in Table 3 are categorical and expressed as percentages. Therefore, it is not possible to calculate standard deviations or 95% confidence intervals for these results.

**R3.1:** Introduction
• The Introduction lacks literature on previous studies.

Answer: We detailed the information of previous studies: “]. In Peru, an evaluation of 17 CPGs published by the Ministry of Health between 2009 and 2014 revealed similar deficiencies [7], including a lack of information about the guideline development team, minimal use of evidence, and unclear recommendation processes [7]. A further study of 31 CPGs developed by the Peruvian Social Health Insurance System (EsSalud) up to December 2015 reported gaps in evidence-based frameworks and inconsistent reporting of recommendations [8].”

**R3.2:** Introduction

• Additional background and explanation on what is considered as an ideal/standard CPG and what is the standard content of CPGs would be helpful to provide additional context. Also, it would be helpful if the authors specify what methodological deficiencies are usually observed in local CPGs.

Answer: Thanks for your comment. We added the following information: “A trustworthy CPG adheres to these standards that emphasize key components including clearly defined clinical questions, systematic literature searches, the use of a grading system for evidence, explicit criteria for formulating recommendations, external review, and a structured process for updates [1,2].”

**R3.3:** Methods
• Page 6, Line 80: Any rationale for considering 2018 as the start year for assessing the CPGs when the standardized methodology document from Ministry of Health was established in 2015?

Answer: Thanks for your comment. We considered it reasonable that, although the new standards were established in 2015, they would begin to be applied starting in 2016, allowing time for training and adaptation. Therefore, the first guidelines fully developed under these standards would likely be published from 2018 onward, making this a more appropriate starting point for our analysis.

**R3.4:** Methods

• Was the literature search not conducted in other search engines including PubMed, SCOPUS etc.?

Answer: Thanks for your comment. In Peru, clinical practice guidelines are not required to be published as journal articles. When they are published in this format, they often present only a summary (a brief justification for each recommendation) of the guideline content rather that the complete document. Therefore, we prioritized searches on local websites, Google, and Google Scholar, where full versions of the guidelines are more likely to be available. This approach allowed us to access the comprehensive documents necessary for our analysis.

**R3.5:** Methods

• Why were CPGs with partial updates not included? Wouldn’t the partial updates also indicate changes based on the 2015 standardized methodology from Ministry of Health?

Answer: Thanks for your comment. Since the standardized methodology was predominantly implemented from 2018 onward, there have not been frequent updates to previous guidelines published under this framework. We identified only two partially updated guidelines developed by the Social Health Insurance of Peru (IETSI-EsSalud). which are listed in the “S2 Table: Table of excluded CPGs”. The first versions of these guidelines, published in 2018, already followed the GRADE methodology, aligning with the standardized approach. The partial updates focused primarily on updating evidence supporting specific recommendations that could influence their direction (e.g., recommend/suggest or not recommend/suggest), rather than revising the overall methodology. Therefore, we deemed it unnecessary to include these partial updates, as they did not represent comprehensive methodological changes.

**R3.6:** Results
• Any reason the sample size (n) was equal to 85 and not 88 for Tables 2 and 3?

Answer: Thank you for your comment. The sample size (n) in Table 2 and Table 3 is 88, not 85. We have made the necessary corrections. Additionally, we re-verified all the information in the Tables regarding percentage calculations (%), and only two corrections were made in Table 1 and Table 2.

**R3.7:** Discussion
• The Discussion should highlight the implications of this study and how the results would help in bringing positive changes related to CPGs.

Answer: We have added the following information: “Efforts to standardize CPG development in Latin America have employed various strategies, gradually shifting toward evidence-based practices since 2014 [30]. This shift, driven by academic institutions and research groups, has prioritized guideline adaptation, adoption, or de novo development, supported by the creation of methodological manuals [30]. This approach has gained traction across the region, with countries like Brazil [31], Colombia [32], Chile [13], Mexico [5], Argentina [12], and Peru [9] establishing standards for both CPG development and implementation of CPGs [30]. In Peru, the first technical document, issued in 2005, classified evidence using the U.S. Preventive Services Task Force model but lacked robust processes for clinical question formulation or guideline adaptation [33]. The 2015 technical document marked progress by incorporating PICO (Population, Intervention, Comparator, Outcome) questions, systematic reviews, and the GRADE approach [9]. Despite improvements, challenges persist in ensuring the consistent production and implementation of evidence-based CPGs.

Improving CPG management in Peru should focus on enhancing the training of methodologists in evidence synthesis, increasing the integration of economic analysis in decision-making, and ensuring greater consistency in guideline development processes. In the coming years, expected actions include greater collaboration between institutions, strengthening oversight mechanisms, and the continued development of robust training programs for CPG developers. Our study contributes to ongoing discussions on improving CPG quality and implementation in Peru by providing a snapshot of current trends and identifying critical gaps that need to be addressed to enhance the evidence base for healthcare decision-making.”
