# Supplementary material for: Methodological characteristics of Peruvian clinical practice guidelines, 2018 – 2023: A scoping review
Source: PLoS One. 2025 Dec 31;20(12):e0339861. doi: 10.1371/journal.pone.0339861 (PMC12755755; doi:10.1371/journal.pone.0339861)
Supplement: Attachment — Submitted filename: Response_to_reviewers_auresp_2.docx [file pone.0339861.s007.docx]

**PONE-D-24-51671R1**
**Title:** Methodological characteristics of Peruvian clinical practice guidelines, 2018 - 2023: a scoping review

**Response letter**

Dear Editor and reviewers,

Thank you for your kind consideration. In this letter, we proceed to respond to the editor’s and reviewers’ comments.

Sincerely,

Alvaro Taype-Rondan, corresponding author

**Editor’s comments:**

1. In addition to the reviewer response already provided, please state in the limitations of this study your rationale for considering 2018 as the start year for assessing the CPGs and for not including Pubmed, Scopus, or other search engines.

Answer: Thanks for your comment. We added the following information in the Discussion section: “Regarding the selection of 2018 as the starting year for assessing the CPGs, we chose this date because it is likely that the first national CPGs fully developed under the methodological standards established in 2015 (9) were published from 2018 onward. This is based on the understanding that national CPG developers began applying the GRADE methodology in 2016, allowing time for training and adaptation. However, we acknowledge that excluding older CPGs may have influenced the results of our analysis.

Finally, our search strategy primarily relied on Google and publicly accessible local repositories, excluding PubMed, Scopus, and other search engines, as CPGs in Peru are not required to be published as journal articles. Consequently, some CPGs may have been omitted if they were not indexed by Google, if their identification as journal articles in the excluded search engines was faster, or if they were only accessible through institutional repositories or other less visible platforms.”

2. Please discuss more on suggestions to adress methodological issues in Peru, especially on how ESSALUD or Peruvian Health Minister could contribute to improve the elaboration of CPGs.

Answer: Thanks for your comment. We added the following information: “Improving CPG management in Peru requires strengthening the training of methodologists in evidence synthesis, integrating economic analyses into decision-making, and ensuring greater consistency in CPG development. Addressing these methodological gaps will require coordinated efforts from key institutions such as EsSalud and the Ministry of Health of Peru. EsSalud, as the largest social health insurance provider and the institution with the most evidence-based CPGs developed using the GRADE methodology, could establish specialized teams of trained methodologists to oversee the CPG development process and ensure adherence to evidence-based standards. Meanwhile, the Ministry of Health of Peru, could implement regulatory policies mandating the use of SRs and economic evaluations in all national CPGs. Furthermore, both institutions could collaborate with academic centers to develop standardized training programs for CPG developers, promoting the use of structured methodologies and transparent decision-making processes. Another key strategy would be the creation of a centralized repository for CPGs, ensuring accessibility, periodic updates, and methodological consistency across institutions.”

3. Provide examples illustrating how the Peruvian first technical document lacked robust processes for formulating clinical questions or adapting guidelines. Please review the entire document ensuring all authors' comments are objective and highlight the methods used to identify shortcomings, specifying particular flaws where applicable.

Answer: Thanks for your comment. We added the following information: “For example, this document described the methodology as a literature review on the pathology of interest, applying recommendation levels based on study design and findings, without a clear and systematic process for reaching conclusions. Moreover, the adaptation of CPGs lacked a systematic approach, as no explicit criteria were established for selecting international CPGs. Additionally, the methodological process was not required to be explicitly presented; instead, the CPG document was structured as a literature summary covering aspects such as the condition's name, definition, associated risk factors, clinical presentation, diagnosis, auxiliary tests, management, complications, referral and counter-referral, flowchart or algorithm, and references [33].”

**Journal Requirements:**

1. Please review your reference list to ensure that it is complete and correct. If you have cited papers that have been retracted, please include the rationale for doing so in the manuscript text, or remove these references and replace them with relevant current references. Any changes to the reference list should be mentioned in the rebuttal letter that accompanies your revised manuscript. If you need to cite a retracted article, indicate the article’s retracted status in the References list and also include a citation and full reference for the retraction notice.

Answer: We have verified all citations and found no references to retracted papers.

**Reviewers’ comments:**

**R2.1:** However, upon further review, I believe the manuscript could be strengthened by explicitly acknowledging a potential bias in the search strategy. Specifically, as stated in the response: "In Peru, clinical practice guidelines are not required to be published as journal articles. When they are published in this format, they often present only a summary (a brief justification for each recommendation) of the guideline content rather than the complete document." This raises the concern that some relevant documents may not have been retrieved using Google as the primary search platform. There is a risk of missing guidelines that are either not indexed by Google or require access through less visible platforms or institutional repositories.

I recommend adding a discussion point in the limitations section to explicitly address this potential bias and the limitations of the current search methodology. Furthermore, considering more comprehensive and systematic search techniques—such as web scraping or leveraging specialized databases—could be a valuable direction for future research. This approach would enhance the robustness of guideline retrieval and ensure better coverage.

Answer: Thanks for your comment. We added the following information in Discussion section: “Finally, our search strategy primarily relied on Google and publicly accessible local repositories, excluding PubMed, Scopus, and other search engines, as CPGs in Peru are not required to be published as journal articles. Consequently, some CPGs may have been omitted if they were not indexed by Google, if their identification as journal articles in the excluded search engines was faster, or if they were only accessible through institutional repositories or other less visible platforms.”
