# Supplementary material for: Methodological characteristics of Peruvian clinical practice guidelines, 2018 – 2023: A scoping review
Source: PLoS One. 2025 Dec 31;20(12):e0339861. doi: 10.1371/journal.pone.0339861 (PMC12755755; doi:10.1371/journal.pone.0339861)
Supplement: Attachment — Submitted filename: Response_to_reviewers_auresp_3.docx [file pone.0339861.s008.docx]

**PONE-D-24-51671R2**
**Title:** Methodological characteristics of Peruvian clinical practice guidelines, 2018 - 2023: a scoping review

**Response letter**

Dear Editor and reviewers,

Thank you for your kind consideration. In this letter, we proceed to respond to the reviewers’ comments.

Sincerely,

Alvaro Taype-Rondan, corresponding author

**Reviewers’ comments:**

**R4.1:** Potential selection bias toward tertiary-level guidelines
The study appears to be skewed toward clinical practice guidelines (CPGs) developed for tertiary-level care. It is unclear whether this is a reflection of availability or if there was an intentional search strategy targeting this level. The authors should clarify whether their inclusion criteria or sources were likely to miss guidelines developed for primary or secondary care.

Answer: We added the following clarification in the Eligibility criteria section: “No restriction was applied regarding the level of healthcare (primary, secondary, or tertiary) for which the CPGs were intended.” This addition clarifies that our inclusion criteria and search strategy were not designed to target or exclude guidelines based on the level of care.

**R4.2:** Institution-based classification vs. Health sector grouping
The decision to classify guidelines by specific institutions (e.g., IETSI, AUNA) may fragment the analysis and limit broader policy implications. Grouping by major health system sectors—such as social security, Ministry of Health, or private sector—could provide a more comprehensive and policy-relevant perspective.

Answer: Thank you for your observation. We agree that grouping by major health system sectors can be useful for policy analysis. However, in Peru, the development of clinical practice guidelines is highly decentralized. Each institution, whether public or private, operates independently and follows its own processes for guideline development.

For example, IETSI is solely responsible for CPG development within EsSalud (the national social security system). In contrast, the Ministry of Health has at least three separate entities involved: DGIESP (part of the Ministry’s central office), INS (its research branch), and individual hospitals, which vary in methodological capacity. Similarly, private organizations such as AUNA and the Peruvian Society of Neurology develop CPGs through their own independent teams.

Given this institutional heterogeneity, we chose to classify CPGs by developing institutions rather than by sector. This approach more accurately reflects current practices and allows identification of methodological strengths and gaps at the institutional level, differences that might be masked by broader sector-level groupings.

We added the following clarifications to the manuscript:

- At the Introduction section: “Despite the existence of this standard, CPG development in Peru remains decentralized. Different institutions, even within the same sector, develop CPGs independently, with distinct methodological teams and varying capacities. This institutional heterogeneity can influence the methodological quality and consistency of CPGs across the country”.
- At the Data extraction section: “Variables of interest included the year of publication, covered area, developing organization (given that Peruvian health institutions operate with distinct processes and capacities)...” and “CPGs were classified according to the developing institution, rather than by broader health system sectors, to more accurately reflect the decentralized structure and variability of CPG development in Peru.”
- At the Limitations section: “Additionally, while CPGs are often grouped by health system sector (e.g., Ministry of Health, Social Security, or private sector), in this study we classified them by institution to better reflect the decentralized nature of CPG development in Peru. This approach allowed for a more accurate characterization of methodological differences among the various entities responsible for their development.”

**R4.3:** Absence of a critical appraisal of methodological quality beyond grade
While the focus is on GRADE components, the lack of a broader critical appraisal (e.g., AGREE II domains) limits the understanding of the overall methodological rigor of the guidelines. Including or at least discussing this could offer a more balanced assessment.

Answer: Thank you for your suggestion. While we acknowledge the value of AGREE II as a comprehensive tool for appraising guideline quality, our study focused on specific methodological characteristics, particularly those related to the use of GRADE. This focus aligns with the Peruvian guideline development manual, which explicitly recommends using GRADE to assess the certainty of evidence and formulate recommendations.

We believe that AGREE II, by combining multiple domains, including reporting quality and editorial Independence, may dilute the focus on core methodological aspects. Our intention was not to provide a broad appraisal, but rather to identify and describe concrete methodological shortcomings that may compromise the reliability of the recommendations.

In addition, as highlighted in a recent systematic review [38], AGREE II presents several limitations, such as the lack of guidance for the two overall assessments, the subjectivity in scoring, and the equal weighting of all items regardless of their relevance. These issues reduce its precision for detailed methodological evaluations. We have clarified this rationale in the limitations section of the manuscript: “Second, we did not use quality assessment tools such as AGREE II because our focus was on specific methodological aspects, particularly those related to GRADE, in line with the Peruvian guideline development manual, rather than an assessment of overall quality. AGREE II provides a broad evaluation framework, but its combination of multiple domains and reported limitations, such as subjective scoring and lack of weighting [38], may reduce its utility for focused methodological analysis.”

38. Hoffmann-Eßer W, Siering U, Neugebauer EA, Brockhaus AC, Lampert U, Eikermann M. Guideline appraisal with AGREE II: Systematic review of the current evidence on how users handle the 2 overall assessments. PLoS One. 2017;12(3):e0174831. doi: 10.1371/journal.pone.0174831.

**R4.4:** Lack of contextual data on developers' capacities
The authors attribute deficiencies to limited methodological capacity but do not provide data to support this claim. Information on the availability of trained personnel, institutional mandates, or access to GRADE training would strengthen the interpretation.

Answer: In the manuscript, we now discuss how limited methodological capacity, including insufficient training in GRADE, has been identified as a major challenge in guideline development in other countries.

We have clarified this in the discussion section: “This shortfall may be attributed to limited methodological capacity, particularly a lack of trained methodologists in GRADE methodology, as reported in other countries in the region, such as Brazil, where despite centralized process for CPG development, there is a persistent shortage of qualified human resources and limited investment in capacity-building for CPG development [23]. Similar capacity constraints have also been observed across Latin America. For instance, a regional analysis of CPGs published between 2011 and 2017 found that many were developed with limited technical and institutional support, insufficient training in evidence-based methodologies, and minimal application of GRADE, compromising both methodological rigor and transparency [24].”

Also, we added the following information: “This gap may reflect structural limitations, such as a shortage of trained personnel in health economics, the absence of clear institutional methodological CPGs for economic evidence, and the lack of structured economic data from Peruvian healthcare services. These challenges are not unique to Peru and have been reported in other countries, such as Brazil, where the need to invest in capacity-building and methods development for economic evaluation in CPGs has also been emphasized [22] Furthermore, insufficient training and fragmented institutional processes have been identified as key obstacles to CPG development across Latin America [24].”

23. Colpani V, Kowalski SC, Stein AT, Buehler AM, Zanetti D, Côrtes G, et al. Clinical practice guidelines in Brazil – developing a national programme. Health Res Policy Sys. 2020;18(1):69. https://doi.org/10.1186/s12961-020-00582-0

24. Cabrera PA, Pardo R. Review of evidence based clinical practice guidelines developed in Latin America and Caribbean during the last decade: an analysis of the methods for grading quality of evidence and topic prioritization. Global Health. 2019;15(1):14. doi: 10.1186/s12992-019-0455-0.

**R4.5:** Need for exploration of policy or regulatory drivers
The manuscript could benefit from examining whether national regulations or incentive structures influence the adoption of GRADE components. This might help explain heterogeneity between institutions or sectors.

Answer: We have now added a paragraph in the Discussion section: “The GRADE system provides a structured, explicit, and transparent method for evaluating evidence certainty and formulating recommendations [20]. Despite national regulations in Peru mandating that CPG development should be based on SRs and utilize the GRADE methodology for recommendations [9], significant variability exists in the CPG development process. This variability may reflect limited enforcement mechanisms and the decentralized nature of CPG development in Peru. Institutions such as EsSalud, which have internal mandates and established technical methodological teams [21], have shown greater adherence to GRADE components. In contrast, other institutions may lack technical capacity or internal regulations that reinforce adherence to these national standards. These regulatory and institutional differences likely contribute to the heterogeneity observed in the use of GRADE components across CPGs.”

9. Ministerio de Salud de Perú. Documento técnico: Metodología para la elaboración de guías de práctica clínica. Lima: Ministerio de Salud de Perú; 2015 [cited 2025 Jan 10]. Available from: https://www.gob.pe/institucion/minsa/informes-publicaciones/314118-documento-tecnico-metodologia-para-la-elaboracion-de-guias-de-practica-clinica

20. Guyatt G, Oxman AD, Akl EA, Kunz R, Vist G, Brozek J, et al. GRADE guidelines: 1. Introduction—GRADE evidence profiles and summary of findings tables. J Clin Epidemiol. 2011;64(4):383–94. https://doi.org/10.1016/j.jclinepi.2010.04.026

21. Instituto de Evaluación de Tecnologías en Salud e Investigación (IETSI). Resolution No. 063-IETSI-ESSALUD-2021: Instruction No. 001-IETSI-ESSALUD-2021 for the development of clinical practice guidelines in EsSalud [Internet]. Lima: EsSalud; 2021 [cited 2025 Jul 5]. Available from: https://www.essalud.gob.pe/ietsi/pdfs/tecnologias_sanitarias/RS_063_IETSI_2021.pdf

**R4.6:** Unclear justification for excluding non-grade guidelines
Excluding guidelines that did not claim to use GRADE may overlook potentially methodologically sound documents. The authors should clarify the rationale and implications of this exclusion on the comprehensiveness of the review.

Answer: We would like to clarify that our inclusion criteria did not require CPGs to explicitly state that they used GRADE. Instead, we included CPGs that reported basing their recommendations on systematic reviews (SRs), as required by Peruvian regulations since 2015 [9].

Our objective was to assess whether CPGs that claimed to use evidence-based methods (i.e., based their recommendations on SRs) incorporated core methodological components aligned with GRADE standards. This approach allowed us to evaluate the rigor and transparency of methodological implementation among CPGs that expressed an evidence-based intent.

We have clarified this rationale in the Eligibility criteria section: “We included Peruvian CPGs that reported basing their recommendations on SRs, an essential component of evidence-based CPG development. This approach allowed us to focus on CPGs with an evidence-based intent and assess whether they incorporated key methodological components aligned with international standards [10-13] and the national regulations for CPG development in Peru [9].”

In addition, we evaluated whether each CPG explicitly mentioned the use of the GRADE methodology (Table 3, 4th variable). Although most CPGs did so (94.3%), a small number of included CPGs based their recommendations on SRs without referring to GRADE. Therefore, our inclusion criterion was not restricted to the use of GRADE, but rather to the presence of SR-based recommendations, which allowed us to explore both the adoption and the use of GRADE components in eligible CPGs.

**R4.7:** Limited Exploration of Guideline Topics and Their Alignment with National burden of disease
The paper does not analyze whether the guidelines reviewed align with national health priorities or the burden of disease in Peru. This would help assess their relevance and strategic focus**.**

Answer: Thanks for your comment. We agree that analyzing the alignment of CPG topics with national health priorities and disease burden is an important area of exploration. However, this was beyond the scope of our study, which focused specifically on assessing methodological characteristics of CPGs, particularly regarding their use of evidence-based components such as SRs and GRADE methodology. Future research could explore the concordance between the content of Peruvian CPGs and the country's epidemiological profile, which would provide valuable insights into the strategic focus and relevance of national guideline development.

**R4.8:** Narrative synthesis could be strengthened with quantitative visualization
A table or heatmap comparing GRADE components across institutions would enhance the clarity and accessibility of the findings, particularly for policymakers and non-specialist readers.

Answer: Thank you for your suggestion. We agree that visual tools can help synthesize complex data. However, we opted to present our findings in detailed percentage-based tables (Tables 2 and 3), which we believe provide more precise and interpretable information, especially for readers who require exact data to inform policy or methodological decisions. A heatmap may obscure the magnitude of differences, particularly for components with low or moderate frequencies. Given the diversity of methodological practices across institutions, we considered that a tabular format would better support the clarity, transparency, and usability of the data.

**R4.9:**  Insufficient discussion of implications for end-user utility
While the absence of Summary of Findings (SoF) tables and EtD frameworks is highlighted, the practical implications for clinicians or decision-makers are not fully elaborated. This connection should be strengthened.

Answer: Thanks for your observation. To address this, we have added a sentence in the Discussion section: “The absence of SoF tables and EtD frameworks in a substantial proportion of CPGs may reduce their practical utility for clinicians and policymakers [20]. SoF tables synthesize the key findings in an accessible format, facilitating transparent and rapid interpretation of the certainty of evidence and magnitude of effects [30]. Likewise, EtD frameworks support decision-makers in adapting recommendations to local contexts by explicitly considering factors such as resource availability, feasibility, equity, and patient preferences [31]. Without these tools, recommendations may lack clarity, contextual relevance, or perceived credibility, potentially limiting their implementation and impact in real-world settings [31].”

20. Guyatt G, Oxman AD, Akl EA, Kunz R, Vist G, Brozek J, et al. GRADE guidelines: 1. Introduction—GRADE evidence profiles and summary of findings tables. J Clin Epidemiol. 2011;64(4):383–94. https://doi.org/10.1016/j.jclinepi.2010.04.026

30. Guyatt GH, Oxman AD, Santesso N, Helfand M, Vist G, Kunz R, et al. GRADE guidelines: 12. Preparing summary of findings tables-binary outcomes. J Clin Epidemiol. 2013;66(2):158-72. https://doi.org/10.1016/j.jclinepi.2012.01.012

31. Alonso-Coello P, Schünemann HJ, Moberg J, Brignardello-Petersen R, Akl EA, Davoli M, et al. GRADE Evidence to Decision (EtD) frameworks: a systematic and transparent approach to making well informed healthcare choices. 1: Introduction. BMJ. 2016;353:i2016. https://doi.org/10.1136/bmj.i2016.

**R4.10:** Need for a forward-looking roadmap or recommendations
The discussion section would benefit from a more detailed set of recommendations or a roadmap for improving CPG development capacity in Peru, including possible institutional partnerships, technical training, or policy reforms.

Answer: Thanks for your comment. We already have included a detailed paragraph in the discussion section where we outline specific strategies, including institutional partnerships, technical training in evidence synthesis and economic analysis, regulatory policy reforms, and the creation of a national repository for CPGs. To better highlight this content, we added the sentence “Based on our findings, we propose a strategic roadmap to improve the development and implementation of CPGs in Peru” at the beginning of the paragraph in the Discussion section.
